# Microenvironmental CTHRC1 has a pro-tumorigenic role in colorectal cancer

**DOI:** 10.18632/oncotarget.28878

**Published:** 2026-05-20

**Authors:** Haylee Duval, Barbara Toomey, Michelle Karam, Tatum Braun, Daniel Fournier, Chloe Grant, Heather Fairfield, Vadim Chepurko, Elena Chepurko, Allyson Schimelman, Brian Nestor, Sergey Ryzhov, Volkhard Lindner, Michaela R. Reagan

**Affiliations:** ^1^Center for Molecular Medicine, Maine Health Institute for Research, Scarborough, ME 04074, USA; ^2^Graduate School of Biomedical Science and Engineering, University of Maine, Orono, ME 04469, USA; ^3^The Roux Institute at Northeastern University, Portland, ME 04101, USA; ^4^Tufts University School of Medicine, Boston, MA 02111, USA; ^5^Department of Biological Sciences, University of Southern Maine, Portland, ME 04103, USA

**Keywords:** Cthrc1, CRC, colorectal cancer, subcutaneous tumor model, immune analysis

## Abstract

Collagen triple helix repeat containing 1 (CTHRC1) is a secreted protein that has previously been explored for its role in tissue remodeling and cancer. However, its function in the tumor microenvironment (TME) remains poorly understood, despite its known expression in tumor-associated stroma. Colorectal cancer (CRC), a malignancy characterized by extensive stromal involvement, poses an excellent opportunity to investigate this gap. Here, we provide the first evidence that host-derived CTHRC1 drives colon cancer progression, with this effect consistently observed across three independent cohorts. Specifically, when injected with CRC cells, *Cthrc1* null (global knockout, KO) mice develop significantly smaller and less dense tumors compared to wild-type (WT) mice. Additionally, median survival increased approximately 2.5-fold in *Cthrc1* KO mice, from 28 days post-inoculation in WT (*n* = 10) to 69 days in CTHRC1-deficient mice (*n* = 10), suggesting CTHRC1 promotes tumor growth within the TME. Immune cell profiling revealed differences in the composition of tumors and spleens of these mice; specifically, *Cthrc1* KO mice exhibited an increased percentage of CD3^+^ T cells in both tumors and spleens and decreased Gr-1+ myeloid cells in the spleen, compared to WT, indicating an immunoregulatory role for CTHRC1 in CRC. These results identify CTHRC1 as a key driver of CRC that may suppress the immune system, allowing for easier immune evasion by tumor cells, highlighting CTHRC1 as a potential new target for therapy.

## INTRODUCTION

Colorectal cancer (CRC) is the third leading cause of cancer-related deaths in both men and women, with a projected 154,270 new cases, accounting for approximately 8% of all new cancer diagnoses in the United States in 2025 alone. Notably, 1 in 24 Americans are estimated to develop invasive CRC in their lifetime, and the prevalence of CRC is rising among adults under 55 years of age [1]. This shift suggests a changing CRC landscape and highlights the need to further understand the mechanisms underlying CRC and the development of better therapies to improve patient outcomes.

Collagen triple helix repeat containing 1 (CTHRC1) is an inducible, 28 kDa glycosylated secreted protein that is detectable in human circulation and is basally expressed by osteoblasts, osteocytes, neurons in the paraventricular and supraoptic nucleus of the hypothalamus, and episodically by fibroblasts following tissue injury or in cancer-associated stroma [2–4]. CTHRC1 contains twelve G-X-Y repeats that facilitate trimer formation, and a propeptide-encoding N terminus that, when cleaved, activates the protein [2, 4]. Most circulating CTHRC1 exists in its cleaved form, which can increase glycolysis and decrease the respiratory exchange ratio in mice [2], enhance cell migration, and regulate ECM synthesis and organization [4, 5]. Since CTHRC1 was first found to be expressed by fibroblasts in balloon-injured arteries in 2005 [4], its roles in a variety of physiological processes have been established, including metabolism [2, 6, 7], bone and joint homeostasis [8, 9], tissue remodeling and ECM synthesis and organization [4, 5, 10], and oncogenesis [3, 11, 12].

Many studies have identified CTHRC1 as a biomarker for metastasis and poor prognosis in cancers including breast [12, 13], head and neck, kidney, liver, lung, stomach, endometrial [14], and CRC [15]. However, many of these studies used bulk RNA-sequencing to identify *CTHRC1* expression, leaving the identity of the cell type expressing *CTHRC1* in the tumor unknown. CTHRC1-positive cancer-associated fibroblasts (CAFs) have been shown to modulate macrophage polarization to the M2 lineage in the tumor microenvironment (TME), which is characterized by anti-inflammatory, angiogenic, and immunosuppressive properties [12, 16]. Using commercially available antibodies that have not been rigorously validated some groups have claimed tumor cells express CTHRC1. However, in our own studies we detected CTHRC1 expression only in CAFs [3], and others agree that CTHRC1 is expressed by CAFs and is a key regulator of CRC progression [15]. In the present study, we investigated the role of host-derived CTHRC1 in CRC progression using a rigorously created and validated CTHRC1 global knockout (KO) mouse and a monoclonal antibody that has been thoroughly validated for specificity to CTHRC1 (clone Vli55) [3]. This antibody has been used to demonstrate that CTHRC1 is specifically and highly expressed by activated stromal cells in the TME of many cancers including gastric, breast, endometrial, pancreatic, kidney, lung, skin, and colon, but is not expressed by the malignant cells [3]. Currently, the precise function of stromal-derived CTHRC1 remains unknown and the consequences of inhibiting CTHRC1 in the TME on tumor outcomes have never been assessed. Additionally, enhanced understanding of how CTHRC1 may promote an immunosuppressive environment could influence future CRC treatments. Thus, we have herein tested the hypothesis that removal of CTHRC1 from the TME will reduce CRC tumor growth and increase anti-tumor immune responses.

## RESULTS

### Human colorectal tumor cells and MC38 cells do not express CTHRC1

**Figure 1:**
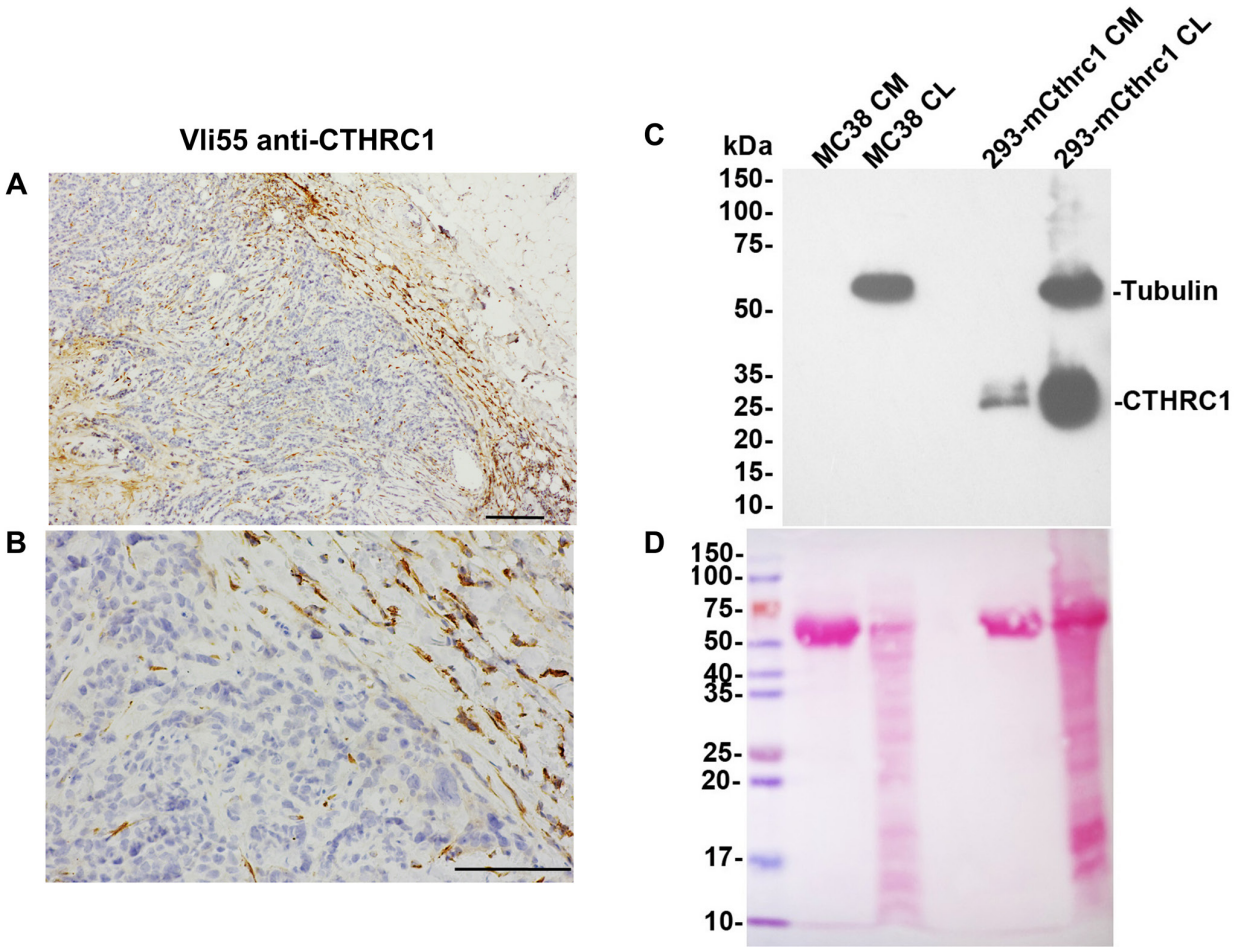
CTHRC1 is not detected in human colorectal tumor cells or MC38 cells. CTHRC1 immunostaining of human colorectal tumor sections with VLi55 at 50X (**A**) and 200X (**B**) magnification shows no CTHRC1 protein present in tumor cells (stained blue). Positive dark brown staining is present in fibroblasts in the stroma surrounding and infiltrating the tumor. Scale bar (A) 200 μm and (B) 100 μm. (**C**) CTHRC1 protein was not detected in MC38 cells in either conditioned medium (CM) or cell lysates (CL) by Western blot analysis using VLi55 anti-CTHRC1 antibody recognizing the completely conserved C terminus of CTHRC1. CTHRC1 is detected in CM and CL from HEK293T cells transfected with an expression construct encoding mouse Cthrc1. Molecular weights are shown in kDa on the left. No bands corresponding to the expected 28 kDa CTHRC1 protein were observed in MC38 samples in the Western blot, even though proteins were present in the lanes. Tubulin staining is used as a loading control for CL samples. (**D**) Ponceau S staining of the same membrane confirms protein presence in each lane. A strong band at ~67 kDa is observed in samples of conditioned medium, which is consistent with albumin as part of the fetal bovine serum present in the culture medium.

In the literature, there is debate whether the tumor cells themselves express CTHRC1, or only the activated cancer-associated fibroblasts (CAFs), which can make up a large portion of solid tumors. We have performed CTHRC1 immunohistochemistry (IHC) on multiple human tumor samples from our MaineHealth Institute for Research (MHIR) Biobank and consistently observed that tumor cells are negative for CTHRC1, while CAFs are strongly positive (Figure 1A, 1B, Supplementary Figure 1A–1I). We have also observed positive CTHRC1 expression in dermal fibroblasts during skin wound healing in a representative wild type (WT) mouse at days 5 and 8 post-injury, while dermal fibroblasts from a representative *Cthrc1* global knockout (KO) mouse at day 5 post-injury lack detectable CTHRC1 expression (Supplementary Figure 1J–1L).

We also performed Western blot analysis of the MC38 cells used in this study and found that they do not produce CTHRC1, either intracellularly (cell lysate; CL) or secreted into the medium (conditioned media; CM). MC38 cells showed no CTHRC1 protein expression, whereas positive control samples of *Cthrc1*-transfected cells showed CTHRC1 at the expected molecular weight (Figure 1C). Ponceau S staining of the same blot confirmed that protein was present in each lane (Figure 1D).

### Knocking out *Cthrc1* reduces subcutaneous tumor burden and increases mouse survival

**Figure 2:**
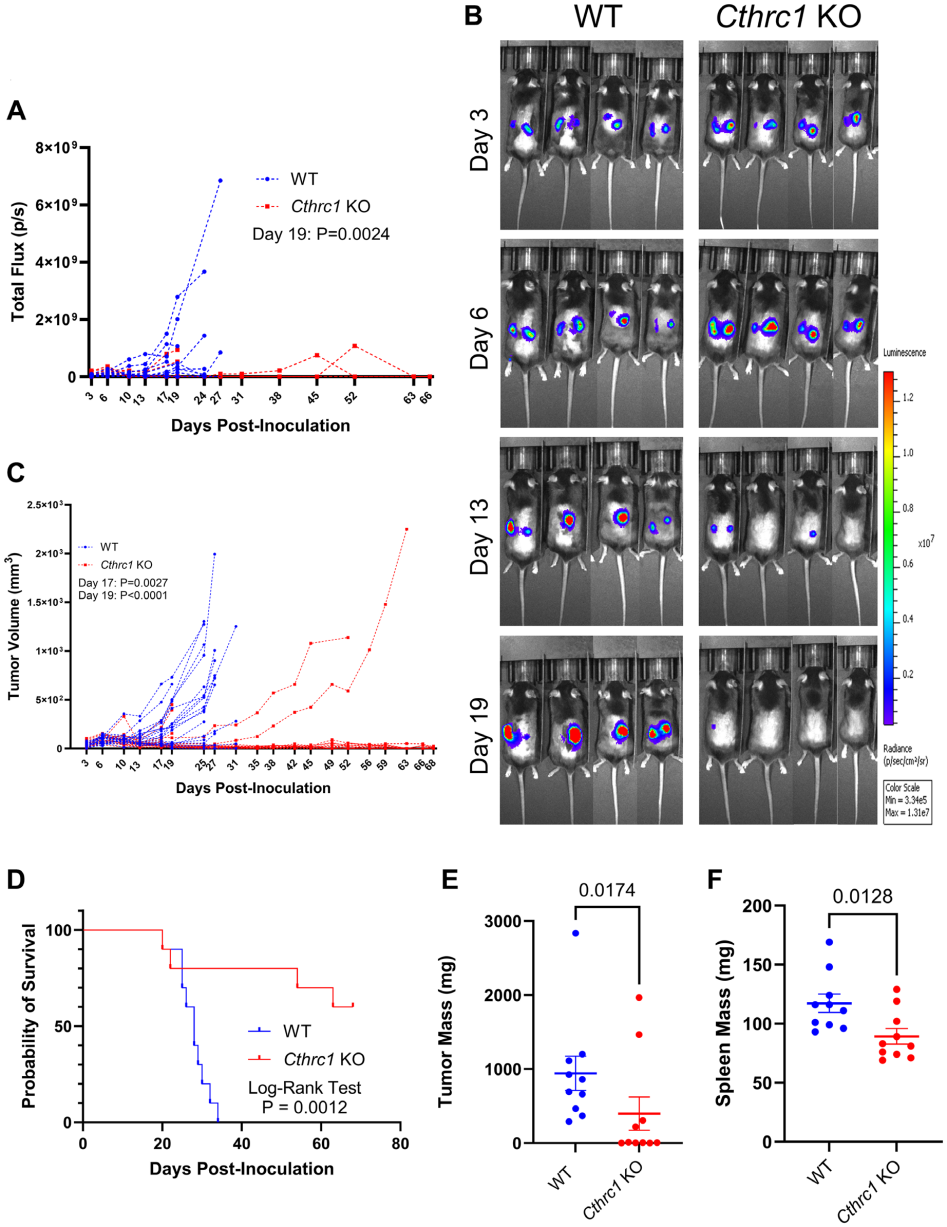
Knocking out *Cthrc1* leads to decreased CRC tumor burden and increases survival. Graph (**A**) shows longitudinal BLI following MC38^Luc+/RFP+^ cell inoculation with significance determined by two-way ANOVA through Day 19, the final day all mice were alive. Panel (**B**) shows representative BLI images of WT and *Cthrc1* KO mice at Days 3, 6, 13, and 19 post-inoculation, with a luminescence scale of 3.34 × 10^6^–1.31 × 10^7^. The representative mice exhibited the smallest deviation from cohort averages. Graph (**C**) shows individual measurements of tumor volume over time. Statistical significance was determined by two-way ANOVA through Day 19, the last day all mice were alive. Panel (**D**) shows a Kaplan-Meier survival curve determined by endpoint criteria as described in Methods, with statistics determined by Log-Rank Test. Graphs of post-mortem (**E**) tumors and (**F**) spleen weights show significant differences between WT and *Cthrc1* KO mice, as determined by Mann-Whitney and unpaired *t*-tests, respectively. (*n* = 10 per group).

To determine if host-derived CTHRC1 contributes to CRC tumor growth and mouse survival, we conducted an *in vivo* study tracking tumor growth with BLI and survival using Kaplan-Meier analysis (Figure 2 and Supplementary Figure 2). *Cthrc1* KO mice had reduced tumor burden at Day 19 post-inoculation by longitudinal BLI compared to WT mice (Figure 2A and Supplementary Figure 2A). This was a result of *Cthrc1* KO mice having either greatly delayed/slowed/absent tumor growth, or tumor growth and then regression, which can be clearly visualized in some mice when comparing days 6 to 13 (Figure 2B). This difference in tumor burden was confirmed by volumetric tumor measurements (Figure 2C and Supplementary Figure 2B). A correlation between BLI-quantified tumor burden and caliper-measured tumor volume is shown in Supplementary Figure 2C. When mice were individually monitored and euthanized upon meeting survival endpoint criteria, *Cthcr1* KO mice survived significantly longer than WT mice, with 60% of *Cthrc1* KO mice never reaching survival endpoint criteria by end of study (Day 69) (Figure 2D). Despite these differences in time of euthanasia, we still compared post-mortem tumor and spleen masses. Surprisingly, these were also reduced in *Cthrc1* KO mice (Figure 2E, 2F). No significant weight differences were seen between WT and *Cthrc1* KO mice, within sexes, (Supplementary Figure 2D), and no adverse effects of *Cthrc1* KO were observed.

### Knocking out *Cthrc1* consistently reduces subcutaneous tumor burden and induces a more active anti-tumor immune microenvironment

**Figure 3:**
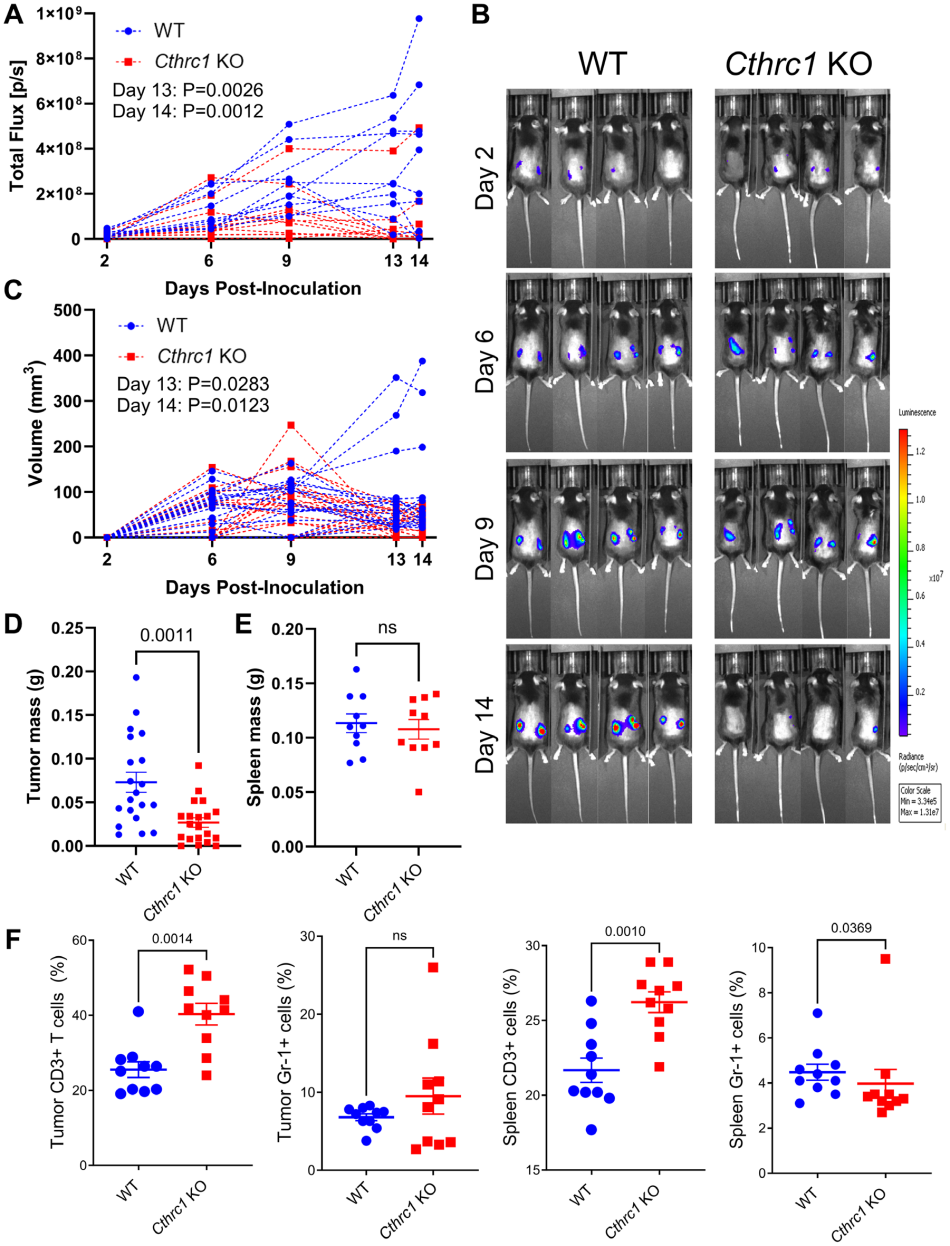
Knocking out *Cthrc1* in mice leads to decreased CRC tumor burden and reduces immunosuppression. (**A**) Shows the longitudinal total flux (p/s) by BLI of individual WT and *Cthrc1* KO mice, each line representing the average total flux between the two tumors on individual mice. Statistics determined by two-way ANOVA. Panel (**B**) shows representative BLI images of WT and *Cthrc1* KO mice at Days 2, 6, 9, and 14 post-inoculation, with a luminescence scale of 3.34 × 10^5^–1.31 × 10^7^. Representative mice were chosen based on the smallest deviation from cohort averages. (**C**) Depicts individual tumor volumes over time as measured by electronic calipers, with left and right tumors graphed individually. Panel (**D**) shows individual tumors weights post-mortem. Statistics determined by Welch's correction for unpaired *t*-tests. Graph (**E**) depicts spleen mass at endpoint, with statistics determined by unpaired *t*-test. Panel (**F**) shows immune cell analyses, specifically the percentage of CD3+ T cells and Gr-1+ myeloid cells from tumors and spleens of WT and *Cthrc1* KO mice, analyzed by Mann-Whitney tests. All data represent mean ± SEM. (*n* = 10 per group).

In an effort to directly compare time-matched tumor and spleen tissues between groups, we then conducted an endpoint study (Figure 3). By Day 13 post-inoculation, *Cthrc1* KO mice exhibited significantly less BLI-quantified tumor burden compared to WT mice (Figure 3A and Supplementary Figure 3A). *Cthrc1* KO mice in this cohort exhibited a tumor regression trend similar to that observed in the survival study, which occurred between Days 9–13, while tumors in WT mice kept growing. This phenomenon was clearly visible on Day 14 in representative BLI images (Figure 3B). Tumor regression in *Cthrc1* KO mice was further captured by tumor volumes measured with electronic calipers (Figure 3C and Supplementary Figure 3B), which also confirmed significantly less tumor burden in *Cthrc1* KO mice by Day 13. Overall reduced tumor volume in the *Cthrc1* KO cohort was further validated by post-mortem tumor weights, which confirmed that tumors from *Cthrc1* KO mice weighed significantly less than those from WT counterparts (Figure 3D). No significant differences were detected in spleen weights or sex-matched body weights between groups (Figure 3E and Supplementary Figure 3C). Further analysis of tumors and spleens collected at sacrifice investigated immune cell differences between groups (Figure 3F). Compared to WT mice, *Cthrc1* KO mice exhibited an increased percentage of CD3^+^ T cells in both tumors and spleens, and decreased Gr-1^+^ myeloid cells in the spleen, indicating a potential inhibitory role for CTHRC1 in immunosurveillance in CRC. The flow cytometry gating strategy for these populations are described in Supplementary Figure 3D, 3E.

To confirm for a third time that *Cthrc1* KO mice are protected from CRC, we repeated the endpoint study using an additional independent cohort (Supplementary Figure 4), which again demonstrated that *Cthrc1* KO mice have reduced tumor burden compared to WT and exhibit tumor regression between Days 7–11 (Supplementary Figure 4A–4D). No significant differences were detected between groups in spleen weights, either post-mortem or longitudinally (Supplementary Figure 4E, 4F), nor in sex-matched body weights throughout the study (Supplementary Figure 4G).

**Figure 4:**
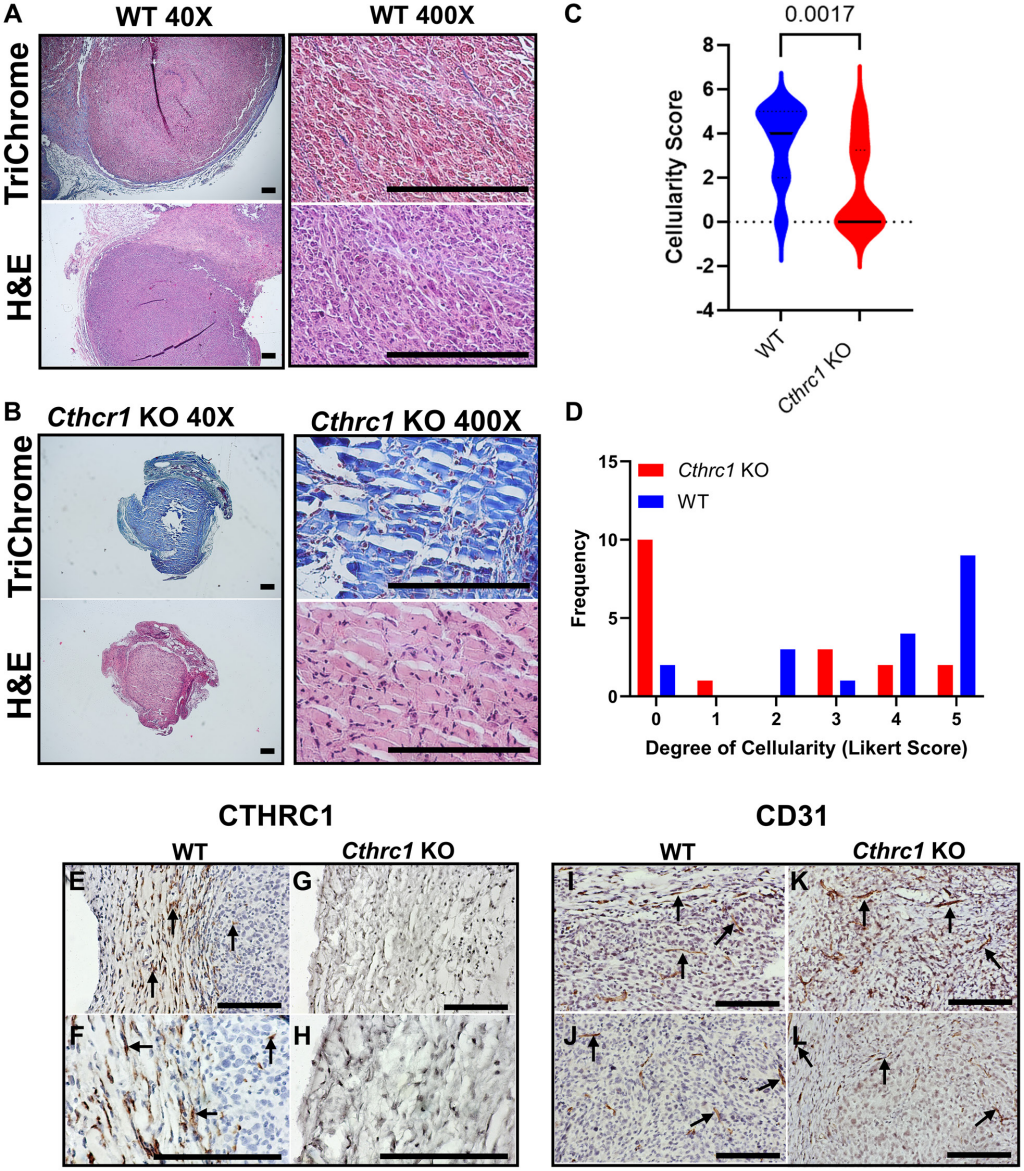
Histological staining reveals *Cthrc1* KO alters tumor microarchitecture and cellularity. Histological sections of mouse tumors from the endpoint study are shown. Tumors from WT and *Cthrc1* KO mice at Day 14 post-inoculation were stained with Trichrome (top) and Hematoxylin and Eosin (H&E; bottom). Representative tumors from (**A**) WT and (**B**) *Cthrc1* KO mice are shown at 40X (left) and 400X (right) magnification. Scale bar, 200 μm. Tumor cellularity per histological sample was scored using a Likert scale as described in Methods and are shown as (**C**) violin and (**D**) histogram plots (*n* = 18 WT, *n* = 19 *Cthrc1* KO). Because the sample size is small and the scores are not normally distributed, differences in cellularity between WT and *Cthrc1* KO tumor samples were assessed using a Mann-Whitney *U*-test. Vli55 immunohistochemical staining of tumor sections at 200X and 400X magnification for (**E**, **F**) WT and (**G**) *Cthrc1* KO mice shows the fibrous capsule and tumor tissue, with positive staining for CTHRC1 only in WT tumors. (**H**) A stained regressed tumor from a *Cthrc1* KO mouse shows residual matrix, few fibroblasts, and no CTHRC1. Scale bar, 200 μm. CD31 immunostaining of tumor sections at 200X magnification shows positive vessel-like structures in stroma and tumors of both (**I**, **J**) WT and (**K**, **L**) *Cthrc1* KO mice. Positive staining (dark brown) is marked by arrows.

### Histological staining reveals *Cthrc1* KO alters tumor microarchitecture and cellularity

To further evaluate tumor morphology in *Cthrc1* KO and WT mice, we employed Trichrome and Hematoxylin and Eosin (H&E) histological staining and IHC analysis of CTHRC1 and CD31 expression on subcutaneous tumors collected at Day 14 post-injection (Figure 4). Trichrome and H&E staining of representative WT (Figure 4A) and *Cthrc1* KO (Figure 4B) tumors showed abundant collagen matrix and paucity of cells in the tumors from *Cthrc1* KO mice. Presumably, the residual acellular extracellular matrix reflects the prior presence of tumor cells. Likert scale quantification of hematoxylin-stained cells revealed significantly reduced cellularity in tumors from *Cthrc1* KO mice compared to WT (Figure 4C, 4D). CTHRC1 staining with Vli55 demonstrated CTHRC1 mainly in the tumor stroma of WT mice, with some positive staining of presumed fibroblasts within the tumor mass close to the fibrous capsule (Figure 4E, 4F). The absence of CTHRC1 in both stroma and tumors of *Cthrc1* KO mice supports the hypothesis that host-derived CTHRC1 is responsible for tumor progression (Figure 4G, 4H). Vascularization of tumors was assessed by CD31 immunostaining (Figure 4I–4L) and no apparent difference between *Cthrc1* KO mice and WT mice was noted.

## DISCUSSION

The studies herein demonstrate a specific role of non-tumor cell (microenvironmental) CTHRC1 in the development of CRC. Mined clinical data had previously associated CTHRC1 with certain cancers [12–15], including CRC, which has a dependence on stroma [17], where CTHRC1 is expressed. Using highly specific CTHRC1 antibodies, we found that tumor cells in human CRC samples and the MC38 CRC cells *in vitro* do not produce CTHRC1 protein. Immunohistochemical staining for CTHRC1 in KO mice was negative in all samples indicating that CTHRC1 is not made by MC38 tumor cells *in vivo* and is host-derived. Comparing subcutaneous CRC growth between immunocompetent C57BL/6 and global *Cthrc1* null mice revealed significant tumor regression in *Cthrc1* KO mice across three independent cohorts. Immune profiling revealed that *Cthrc1* KO mice have increased T cell percentages in both tumors and spleens, and decreased splenic Gr-1^+^ myeloid cells, suggesting both stromal and circulating CTHRC1 may contribute to immunosuppression in WT mice. Because the Gr-1^+^ population includes myeloid-derived suppressor cells (MDSCs), which inhibit T cell activation and proliferation [18], reduced systemic Gr-1^+^ myeloid cells in *Cthrc1* KO mice may reduce myeloid-mediated T cell suppression and contribute to CRC protection. Variability in tumor progression/regression in individual mice could be related to innate differences in splenic immune composition, which warrants further study.

The exact mechanism by which *Cthrc1* KO mice are protected from CRC is unknown and likely multifaceted, and a more detailed investigation into the effects of CTHRC1 on immune cell regulation is an important next step to understanding this protection. We have a very good understanding of the timing of CTHRC1 expression in response to fibroblast activation occurring after myocardial infarction, skin incisional wounding, and arterial injury in mice. In these acute injury models, fibroblasts start to express CTHRC1 as determined by immunostaining within two days, with CTHRC1 expression increasing over the following days until a gradual decline in expression is observable from two weeks on with very few fibroblasts expressing CTHRC1 four weeks post-injury [5, 10]. In all cohorts of the present study, there was initial growth of tumors, but significant tumor regression was seen after approximately 9 days in the *Cthrc1* KO mice, with most tumors in WT mice continuing to grow. If induction of CTHRC1 by the presence of tumor cells follows a similar timeline, this would explain the timing of immunosuppression and promotion of tumor growth in WT mice. Although universal tissue repair processes that result in complete repair likely follow a similar time course of CTHRC1 expression, in the context of expanding tumor growth, we anticipate that stromal fibroblast activation is ongoing, resulting in sustained CTHRC1 expression. We were not able to study the exact timing of CTHRC1 expression in these mice, a limitation to our study. Future time course studies, along with inducible *Cthrc1* KO models (either global or cell type specific), will help determine at what time, and in what cells, the CTHRC1 silencing should occur for the anti-tumor effects.

Another important limitation of the study herein is the subcutaneous nature of this model, in part due to differences in TME immune composition between subcutaneous and orthotopic tumors in colon tissues. Zhao and colleagues [19] demonstrated that, compared to subcutaneous models, orthotopic CRC tumors have stronger anti-tumor immune responses (e.g., increased presence of T cell and B cells, increased NK-cell infiltration, and higher expression of cytokines IL2, IL6, IFNγ and granzyme B), whereas subcutaneous CRC tumors exhibit increased levels of immunosuppressive MDSCs. These differences in immune composition between models have important implications for translation to human patients, particularly in the context of immunotherapies, as immune cell infiltration is known to impact patient outcomes [20]. Given the relatively sparse immune landscape and immunosuppressive TME of the subcutaneous CRC model used in the present study, it is possible that the protection from CRC progression observed in *Cthrc1* KO mice may be even more pronounced in a more immune-infiltrated orthotopic environment. Further considerations when working with a subcutaneous model of CRC include differences in the ECM, which can influence mechanical properties, angiogenesis, and stromal cell interactions [21]. Future studies should therefore assess CTHRC1 function in orthotopic models of CRC.

Many studies have suggested that CTHRC1 promotes angiogenesis [2, 11]. Although there do not appear to be overt differences in the presence of CD31^+^ vessels in tumors between WT and *Cthrc1* KO mice, solid tumors often have aberrant angiogenesis that influences the tumor microenvironment and tumor immune cells [22]. Further study of the mechanisms and timing of CTHRC1’s action on the TME will be critical to fully understanding its role in tumor promotion. In humans, targeting CTHRC1 may hold great potential as a novel strategy in oncology, but first, we purport, the receptor(s) for it must be identified, and inhibitors to these designed. Overall, we find that elevated CTHRC1 expression in cancer contributes to a dysregulated TME, leading to tumor growth.

## MATERIALS AND METHODS

### Cell culture

MC38^Luc+RFP+^ murine colon cancer cells (ALSTEM; Cat# LRL04) were cultured in DMEM with 4.5 g/L glucose, L-glutamine, and sodium pyruvate (Corning, Cat # 50-003-PB) supplemented with 3.7 g/L sodium bicarbonate (Sigma, Cat # S5761), 10% fetal bovine serum (Gibco, Cat # A52568-01), and 1% penicillin-streptomycin (Anti-Anti; Gibco, Cat # 15240-062). Cells were maintained by weekly media changes and passaging at 37°C with 5% CO_2_. For additional details and validation, see Supplementary Methods.

### *In vivo* studies

All experimental procedures involving mice were approved by the Institutional Animal Care and Use Committee (#2406) of MaineHealth. C57BL/6J (wild type, WT) and Cthrc1^tm1Vli^C57BL/6J (*Cthrc1* KO) global null mice, previously described [6], were bred within the MHIR animal facility.

In all studies, mice received bilateral subcutaneous injections of 5 × 10^5^ MC38^Luc+RFP+^ cells in serum-free DMEM and Matrigel (Corning; Cat# 356237). Tumor growth was monitored by bioluminescent imaging (BLI) and electronic calipers (VWR^®^; Cat# 62379-531) twice weekly. Mice were euthanized if one or both tumors ulcerated or reached 2 cm in diameter, which served as the two defined endpoint criteria.

Three independent cohorts were used: a survival cohort, an endpoint cohort, and a repeated endpoint cohort, each consisting of 20 mice (10 WT and 10 *Cthrc1* KO). The survival and endpoint cohorts included 5 males and 5 females per group. The repeated endpoint cohort included 4 male and 6 female WT mice, and 5 male and 5 female *Cthrc1* KO mice.

Survival mice were inoculated at approximately 10.5 weeks of age and euthanized individually upon reaching an endpoint criterion. Mice that survived to Day 69 post-inoculation were sacrificed at that time. Endpoint mice were inoculated at approximately 8.5 weeks of age and euthanized on Day 16 post-inoculation, when most had reached an endpoint criterion. The repeated endpoint cohort was inoculated at approximately 9 weeks of age and euthanized on Days 18–19 post-inoculation, when most had reached an endpoint criterion. In this cohort, two male *Cthrc1* KO mice and four WT mice (3 male and 1 female) did not survive to the endpoint; all remaining mice were euthanized at that time.

### Immunohistochemistry and microscopy

Subcutaneous tumor composition and CTHRC1 prevalence were assessed by IHC. Tumors were fixed in 10% neutral buffered formalin (NBF) for 24–48 hours, then preserved in either optimal cutting temperature (OCT) compound for frozen sectioning or 70% ethanol for paraffin embedding. Sections were stained with hematoxylin and eosin (H&E), Masson’s trichrome, or antibodies against CTHRC1 or CD31 (Abcam, ab182981) to identify endothelial cells. IHC for CTHRC1 was performed with extensively validated rabbit monoclonal antibody (Vli55, www.mhir.org/antibody) as previously described [6]. Slides were imaged using an Axioskop 40 microscope (Zeiss). Formalin-fixed RFP^+^ tumor tissues embedded in OCT were imaged using an EVOS 5000 Imaging System (ThermoFisher Scientific, AMF5000). Maine Medical Center’s BioBank provided us with deidentified, formalin-fixed, paraffin-embedded tissue blocks of breast, lung, and colon cancer on which CTHRC1 immunohistochemistry was performed with Vli55 as described. Images of representative tumors are shown.

### Immunology and flow cytometry

Single-cell suspensions were prepared from tumors and spleen samples for immune profiling by flow cytometry. Tumor samples were minced and enzymatically digested with collagenase I/dispase II and DNase I for one hour at 37°C. Tumors and spleens were gently pressed though a 40 μm cell strainer and subjected to red blood cell lysis before resuspension in FACS buffer (PBS containing 0.5% BSA and 2 mM EDTA) for staining and analysis. Additional procedural and antibody details are provided in Supplementary Methods.

### Statistical analysis

Single day comparisons were analyzed by unpaired *t*-tests and data are presented as mean ± SEM unless otherwise stated. Longitudinal comparisons were analyzed by two-way ANOVA followed by Šidák’s post hoc test. Images of tumor H&E-stained sections were evaluated using a Likert scale by a blinded investigator to assess cellular density. Scores were assigned as follows: 0 = 0%, 1 = 0–25%, 2 = 25–50%, 3 = 50–75%, 4 = 75–100%, and 5 = 100% dense cellularity.

## SUPPLEMENTARY MATERIALS


