# Supplementary material for: Microenvironmental CTHRC1 has a pro-tumorigenic role in colorectal cancer
Source: Oncotarget. 2026 May 20;17:258–67. doi: 10.18632/oncotarget.28878 (PMC13235751; doi:10.18632/oncotarget.28878)
Supplement: Supplementary file 1 [file oncotarget-26-049725-s001.pdf]

Microenvironmental CTHRC1 has a pro-tumorigenic role in colorectal cancer

SUPPLEMENTARY MATERIALS

In vivo tumor model additional details

The primary investigator was blinded between genotype groups and treatments throughout all studies. Mouse genotypes were confirmed by PCR using the following primers:

Target	Forward (5′→3′)	Reverse (5′→3′)	Product (bp)
Cthrc1 WT	CCACTGGAAACCTCTGGAGTTG	AAGTTCACACAAAGGAAGCCCCGC	209
Cthrc1 KO	GTGTGTTTTGAGGTGTGGTCCC	TGGATGTGGAATGTGTGCGAGG	279

Mice were identified by ear notches and grouped with littermates to reduce incidents of single-housing. During inoculation, mice were sedated via isoflurane (Dechra Veterinary Products; NDC 17033-094-25) and injected subcutaneously with 5 × 10^5 MC38$^{Luc+/RFP+}$ cells suspended in 100 µL of a 1:1 ratio of phenol-red free Matrigel (Corning; Cat# 356237) and serum-free DMEM using 27 G × ½ inch needles (BD; Ref# 305620). Mice were inoculated in a random order and monitored under heat during recovery. Beginning 2-4 days post-inoculation, BLI was preformed twice weekly under isoflurane sedation using a 30-second exposure on an IVIS Lumina LT (Perkin Elmer, Inc., Waltham, MA, USA), 15 minutes after an intraperitoneal (IP) injection of 7.5 mg/mL filter-sterilized D-luciferin (VivoGlo™, Promega). Once palpable, individual tumors were also measured by electronic calipers to calculate tumor volume using the standard formula (L × W × W)/2 as a secondary measure of tumor burden. Individual body weights were recorded throughout each study, and mice were shaved along their backs prior to inoculation and as needed throughout the studies to optimize BLI imaging. At endpoints, euthanasia was performed by either CO_2 inhalation followed by cervical dislocation or isoflurane sedation followed by a ~400 µL IP injection of 2.5% tribromoethanol (Avertin) prior to exsanguination and cervical dislocation. Spleens and tumors were collected, weighed, imaged, and processed for immunohistochemistry (IHC) or immune cell analysis.

Cell culture and validation, protein electrophoresis and western blotting

MC38$^{Luc+/RFP+}$ cells were purchased in 2024 and validated as negative for the following infectious diseases by PCR on 3/24/2024, by Charles River Research Animal Diagnostic Services (Wilmington, MA): Epstein-Barr virus; Hepatitis A, B, and C; Herpes Simplex Virus 1, 2, 6, 7, and 8; HIV-1, -2, -16, and -18; Human cytomegalovirus; Human T-lymphotropic virus; LCMV; Parvovirus B19; SV-40; C. *bovis*; and Mycoplasma. Cells again tested negative for mycoplasma on 7/21/2025 using the MycoAlert® Mycoplasma Detection Kit (Lonza, Cat # LT07-418). Cells used *in vivo* were below passage 10, and cells used *in vitro* were below passage 30.

MC38 cells (ATCC) were grown in 10 cm tissue culture dishes to 70% confluence in DMEM + 10% FBS and 1% penicillin/streptomycin (P/S) in a tissue culture incubator (37°C with 5% CO_2). Media was replaced with serum free DMEM (SF DMEM) with 1% P/S after 24 hours, and cells were grown for another 48 hours. Conditioned media (CM) was collected, spun, and processed for Western blot analysis by the addition of 10% FBS, Laemmli sample buffer (SB), and heating to 95°C for 5 minutes. 20 µl of CM was loaded on 15% SDS PAGE.

CTHRC1 positive control samples were prepared from HEK293T cells transfected with a plasmid containing the gene for mouse Cthrc1. HEK293T cells were seeded on tissue culture plates and grown to 70% confluence in complete DMEM. Cells were transfected with mouse full-length Cthrc1 cDNA using X-tremeGENE™ 9 as the transfection reagent (0.8 mL Opti-MEM I; Gibco) + 8 µg plasmid DNA + 24 µL X-tremeGENE™ 9; Sigma-Aldrich). The following day, the cells were rinsed with PBS, and SF DMEM was added. CM was collected after two days, spun to remove cells, and CM was mixed with 10% FBS, SB, heated to 95°C, and 20 µl of CM was loaded onto the gel.

Cells were collected from each plate after rinsing with PBS by the addition of 0.3 mL RIPA lysis buffer with protease inhibitors (Sigma-Aldrich), scraping into a 1.5 mL tube, sonication, and centrifugation to remove cell debris. Protein concentration was measured with the BCA assay (Pierce™ BCA Protein Assay kit; Thermo Scientific), 10% FBS and SB were added, heated to 95°C, and 100 µg of protein (not including FBS) was loaded on the gel.

Samples were transferred to polyvinylidene difluoride (PDVF) membranes followed by staining with Ponceau S Solution (Sigma) to measure protein levels. Membranes were then washed with 0.1N NaOH followed by H_2O and PBST. Membranes were incubated in 5% nonfat milk in PBS+0.05% Tween20 (PBST) for 30 minutes, and CTHRC1 (VLi55) antibody was added at 0.2 ng/ml overnight at 4°C. For CTHRC1 antibody characterization, see (https://mhir.org/center-for-molecular-medicine/shared-research-facilities/antibody-products/). After rinsing with PBST, anti-rabbit HRP-conjugated secondary IgG was used at 1:5000 and detected with Clarity™ Western ECL Substrate (BioRad). The same membrane was then washed with PBST and incubated with tubulin antibody in 5% milk in PBST (0.2 ng/ml; Sigma, #T5158) overnight at 4°C. After rinsing with PBST, anti-mouse HRP-conjugated secondary IgG was used at 1:5000 and detected with Clarity™ Western ECL Substrate (BioRad).

Immunology and flow cytometry additional details

Tumors were minced into small pieces, suspended in RPMI-1640 media without supplements, and vortexed briefly. Collagenase I/dispase II (1:10; Roche, #10269638001) and DNAse I (1:20; Millipore-Sigma, #4716728001) working solutions were added directly to suspensions and incubated for 1 hour at 37°C with gentle shaking before filtration through a 40 µm cell strainer. Whole spleens were excised, weighed, and gently pressed through 40 µm cell strainers using the plunger end of a 3 mL syringe. Tumor and spleen strainers were rinsed with RPMI-1640, and cells were pelleted by centrifugation at 300 × g for 5 minutes. Red blood cells were lysed using 1X RBC Lysing Buffer (Sigma, #R7757-100ML) according to the manufacturer's instructions and neutralized with 10 mL 1X PBS. Cells were pelleted and resuspended in Miltenyi FACS buffer (PBS containing 0.5% BSA and 2 mM EDTA) for staining and analysis by flow cytometry using 4',6-diamidino-2-phenylindole (DAPI; BioLegend, #422801) to assess viability, and the following anti-mouse antibodies from BioLegend: CD73 (TY/11.8; Cat# 127219), CD49d (MFR4.B; Cat# 103705), CD3ε (145-2C11, Cat# 100319), CD19 (1D3/CD19; Cat# 152409), Gr-1 (RB6-8C5, Cat# 108423), PDGFRa (APA5, Cat# 135905), CD45 (30-F11; Cat# 103113), CD8a (53-6.7; Cat# 100705), CD4 (RM4-4; 116013). CTHRC1 was detected using a monoclonal anti-rat/mouse antibody (catalog number Vli08G09, created in-house, found at https://mhir.org/antibody).

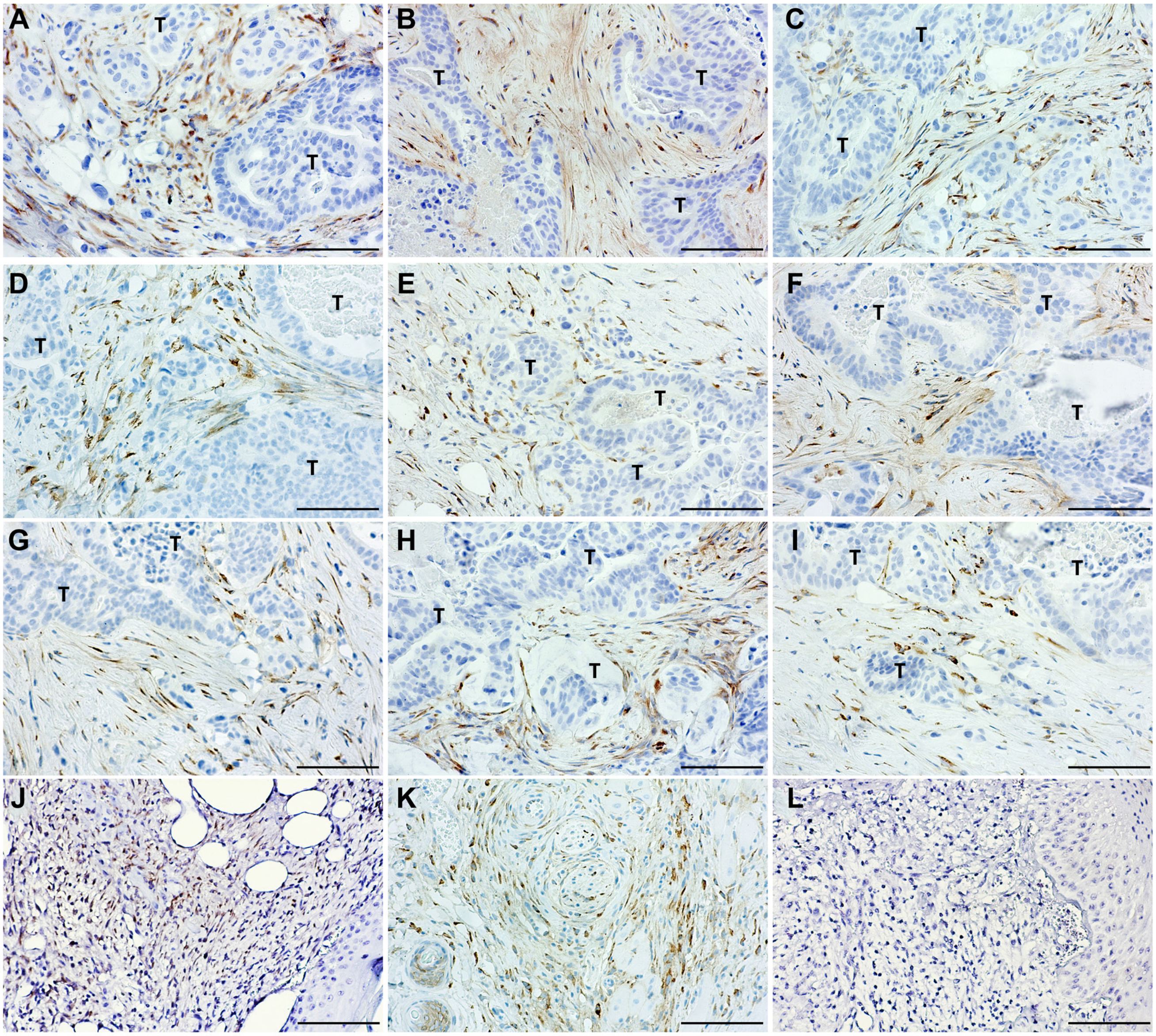

Supplementary Figure 1: Immunohistochemistry for CTHRC1 was performed on three different human lung cancers (**A**–**C**), three human breast cancers (**D**–**F**), and three human colon cancers (**G**–**I**). CTHRC1 is not expressed by tumor cells (T), whereas it is readily detectable in stromal cells (brown). Panels (**J**, **K**) show skin wound healing from a WT mouse at days 5 and 8 post-injury, respectively, with CTHRC1 expression in dermal fibroblasts. Panel (**L**) represents a skin wound from a *Cthrc1* KO mouse at day 5 post-injury, lacking positive CTHRC1 staining. Brown staining represents positive CTHRC1 protein. Scale bars represent 200 µm.

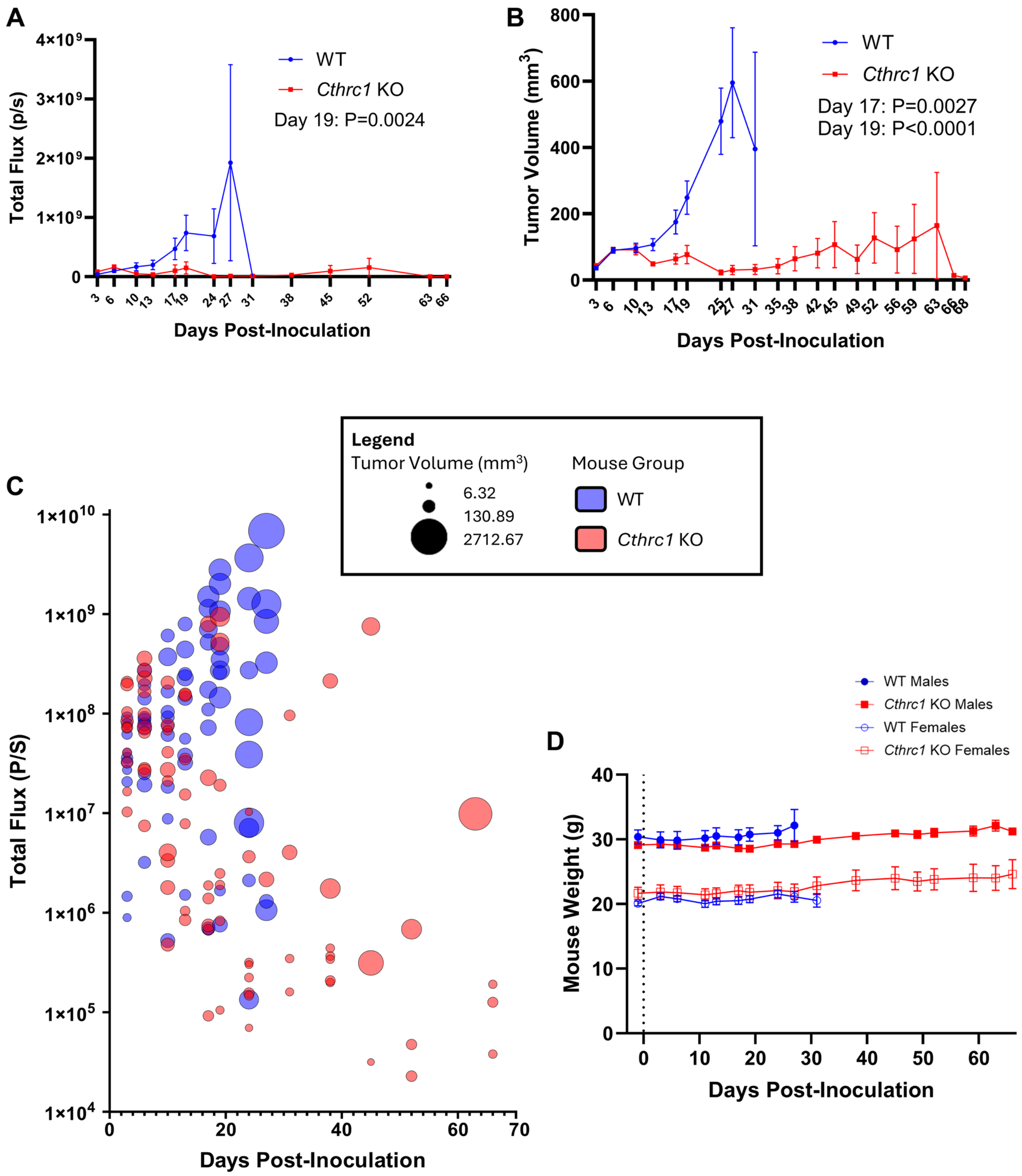

Supplementary Figure 2: Longitudinal measurements in WT and *Cthrc1* KO mice (n = 10 per group). Graph (**A**) shows the average flux from two tumors per mouse through the end of the study. Graph (**B**) shows longitudinal averaged tumor volume by caliper measurements. Statistical significance was determined by two-way ANOVA through Day 19, the last day all mice were alive. Bubble plot (**C**) shows the correlation between BLI- and caliper-quantified tumor burden. Panel (**D**) depicts average mouse weights throughout the study, with the dotted line indicating the day of inoculation. No significant differences between sex-matched genotypes were observed at any time point as determined by Holm-Šídák method for multiple *t*-tests.

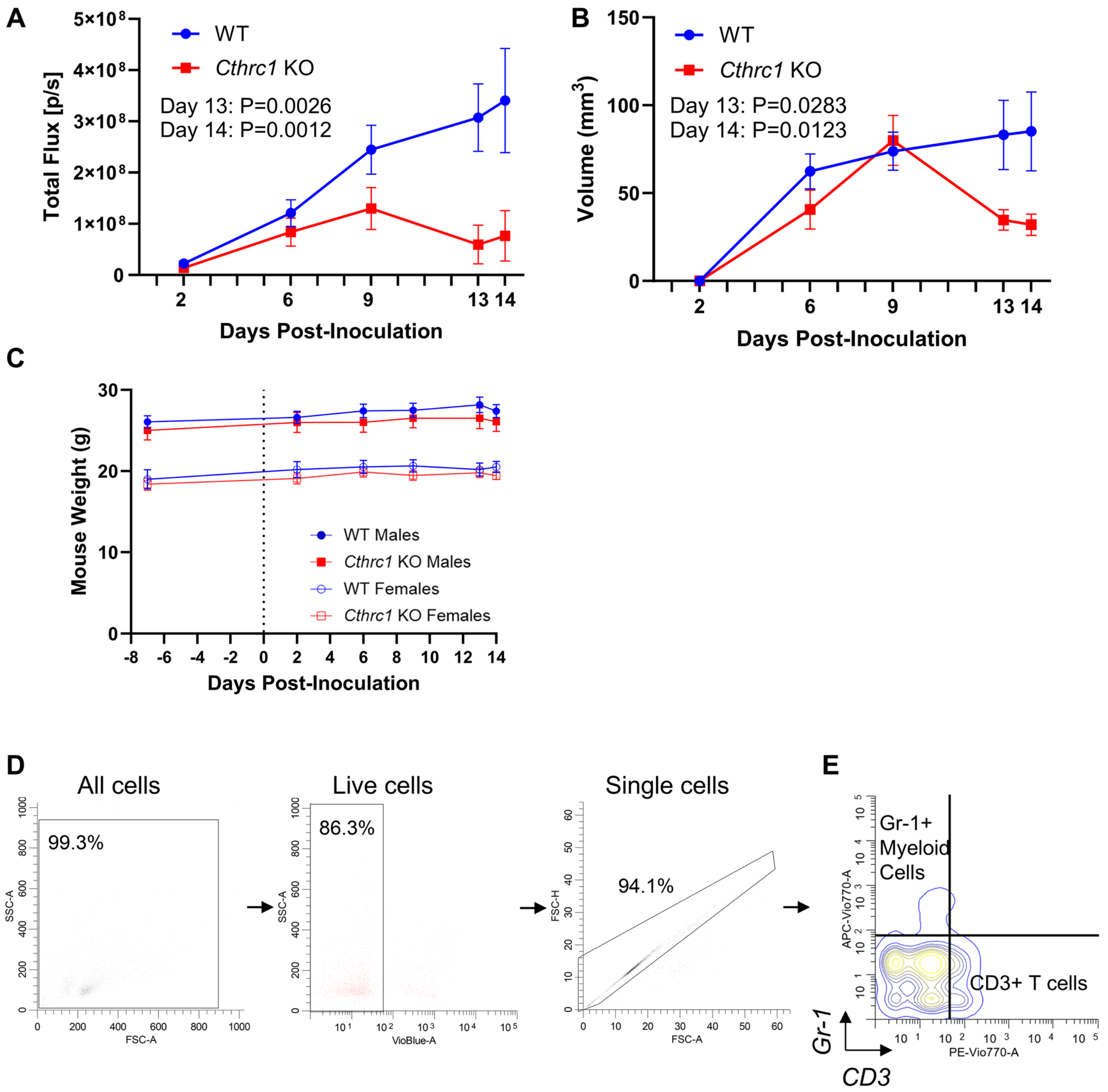

Supplementary Figure 3: (**A**) Shows longitudinal BLI of MC38$^{Luc+/RFP+}$ tumors averaged by group over the 2-week study with statistics determined by two-way ANOVA. Graph (**B**) shows longitudinal averaged tumor volumes via caliper measurements, with statistics determined by two-way ANOVA. (**C**) Shows longitudinal average mouse weights by group. No significance at any timepoint determined by Holm-Šídák method for multiple *t*-tests. (A–C, n = 10 each group; total n = 20). All flow cytometry samples were subjected to (**D**) nest gating strategies to capture (left) all cells, (middle) live (DAPI^{-}) cells, and (right) single cells. Within this final population, (**E**) Gr-1^{+} myeloid cells were identified in quadrant I (Gr-1^{+}, CD3^{-}), and CD3^{+} T cells are identified in quadrant III.

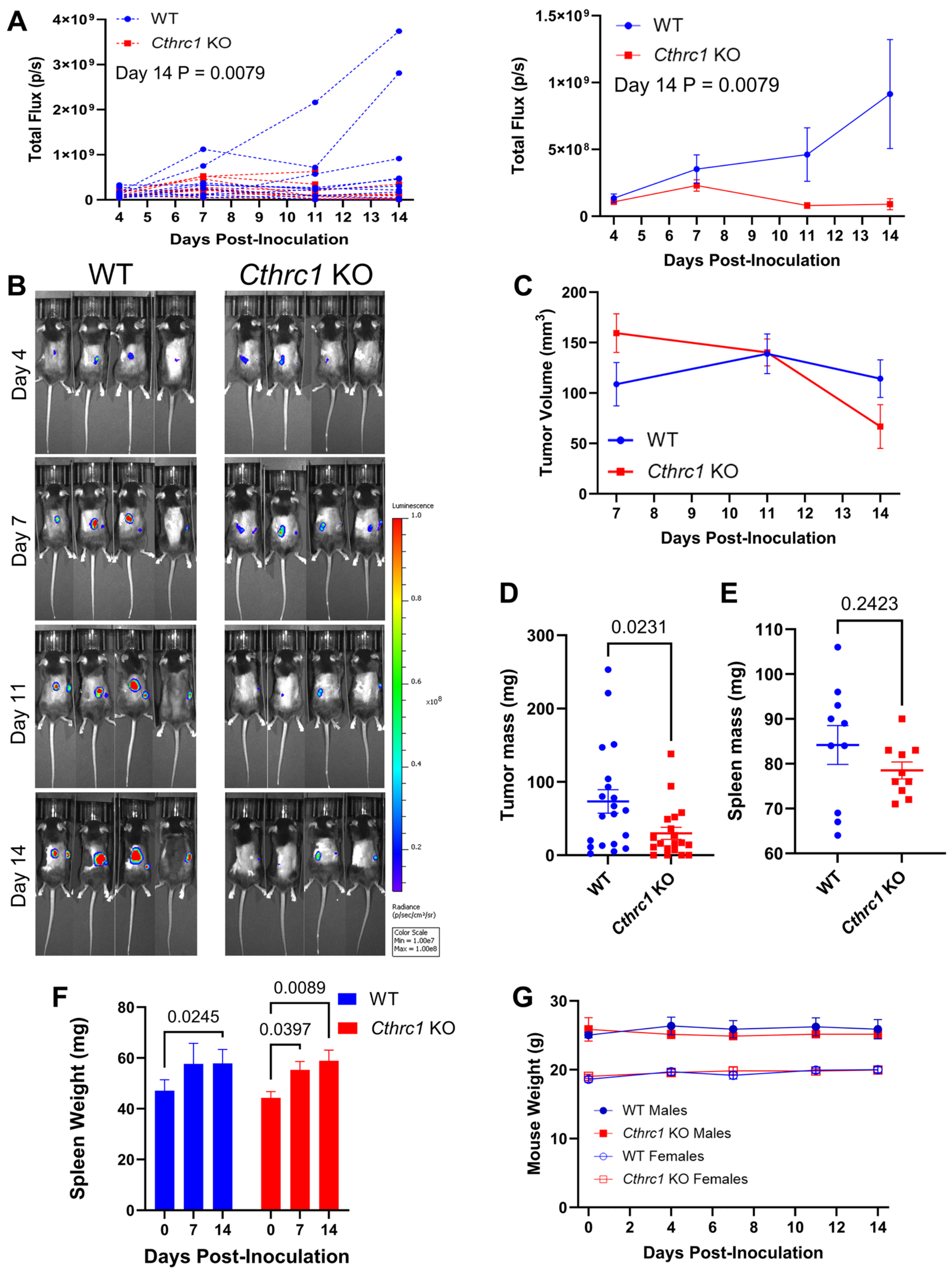

Supplementary Figure 4: Graph (**A**) shows longitudinal BLI after MC38$^{Luc+/RFP+}$ cell inoculation, with the left graph depicting individual mice and right showing cohort averages (n = 10 each group; total n = 20). Statistical significance was determined by Mann-Whitney *U*-test on Day 14, the last day all mice were alive. Panel (**B**) shows representative BLI images of WT and *Cthrc1* KO mice, with a luminescence scale of 1.00×10^7–1.00×10^8. Graph (**C**) shows longitudinal cohort averages of tumor volume by caliper measurements. Panel (**D**) shows tumor mass on the day of sacrifice, with each point representing an individual tumor, compared between WT and *Cthrc1* KO groups. Statistical significance was determined by unpaired *t*-test. Panel (**E**) shows post-mortem spleen mass in WT and *Cthrc1* KO mice, with statistical significance determined by unpaired *t*-test. Graph (**F**) shows longitudinal non-invasive ultrasound measurements of spleen weights in WT and *Cthrc1* KO mice, with mixed-effects analysis by Šídák's multiple comparisons test. Graph (**G**) depicts average mouse weights from before inoculation through Day 14. All data represent mean ± SEM.
